# Two-Stage Intelligent Model for Detecting Malicious DDoS Behavior

**DOI:** 10.3390/s22072532

**Published:** 2022-03-25

**Authors:** Man Li, Huachun Zhou, Yajuan Qin

**Affiliations:** School of Electronic and Information Engineering, Beijing Jiaotong University, Beijing 100044, China; 20111018@bjtu.edu.cn (M.L.); yjqin@bjtu.edu.cn (Y.Q.)

**Keywords:** malicious behavior, statistic model, neural network model, DDoS

## Abstract

5G technologies provide ubiquitous connectivity. However, 5G security is a particularly important issue. Moreover, because public datasets are outdated, we need to create a self-generated dataset on the virtual platform. Therefore, we propose a two-stage intelligent detection model to enable 5G networks to withstand security issues and threats. Finally, we define malicious traffic detection capability metrics. We apply the self-generated dataset and metrics to thoroughly evaluate the proposed mechanism. We compare our proposed method with benchmark statistics and neural network algorithms. The experimental results show that the two-stage intelligent detection model can distinguish between benign and abnormal traffic and classify 21 kinds of DDoS. Our analysis also shows that the proposed approach outperforms all the compared approaches in terms of detection rate, malicious traffic detection capability, and response time.

## 1. Introduction

5G technologies provide ubiquitous connectivity while also addressing the demands of both individual consumers and businesses; however, a tremendous surge of data makes cyber-security a pressing issue, and in order to deal with security threats, various standards organizations including 3GPP, IEEE, and ETSI have been looking into security issues for 5G networks [1].

In 2019, the DDoS attack landscape report [2] indicated that DDoS, a common attack method, is still one of the main threats facing networks, affecting the availability and performance of network services. In 2020, the DDoS attack landscape report [3] pointed out that, with the rapid development of network devices, different varieties of low rate attacks and distributed reflection denial of service attacks are emerging. Thus, it is necessary to upgrade protection measures. An Internet network security monitoring data analysis report [4] summarized the data in terms of malicious programs, vulnerability risk, DDoS attacks, and website security. According to the results, DDoS attacks are still one of the most common malicious behaviors because of their low cost and obvious effect. By controlling multiple computers, DDoS attackers send intensive attack packets to the victim, perform specific malicious behaviors, and consume the victim’s computing resources. There are various ways to form DDoS malicious behaviors. DDoS attackers control machines to build botnets to send large numbers of packets to flood victim hosts. Another possible method of attack is that an attacker will exploit the vulnerability of TCP three handshakes, UDP, or application layer protocols, leading to complex malicious behavior. The malicious behavior includes botnets, low-rate, network layers, DRDoS (distributed reflection denial of service), application layer DDoS, etc. Before launching an attack, the attacker obtains full information about the victim’s application service or the network protocol vulnerabilities. According to their specific vulnerabilities, the malicious behavior is launched. This leads to an end to normal service. Furthermore, the network routers do their best to forward packets that transmit communication protocols, including legitimate and attack packets. The network is a collection of queues, and each queue has a cache area. When the malicious packets are sent constantly, packets fill up the cache area, further increasing the victim congestion and overload. This leads to reduced network performance [5]. As the attack numbers and complexity continue to grow, AI addressing security issues is a new trend; therefore, with the development of 5G networks, a necessary measure is the ability to automatically detect DDoS [6]. 

At present, there are two approaches used to detect DDoS, including statistical analysis and the deep neural network method [7]. By setting an appropriate threshold, statistical methods can identify attacks. Wang et al. [8] proposed the BotMark method, which calculates the flow stability based on the packet length. A single feature is not sufficient to represent the entire flow behavior. The more features there are, the greater the computational load will be. Only a single feature calculates the flow stability, which can result in small computation and a low detection latency. Nevertheless, a single feature can increase false positives. Ghasabi et al. [9] proposed an EWMA (an exponentially weighted moving average mechanism) combined with Jeffrey distance to detect a variety of attacks, while not having the effect on the network. 

We can utilize multiple features to gain flow stability. The flow stability can fully represent the flow characteristics. The exponentially declining weighted moving average method can estimate the flow similarity value. Although the network traffic will change dynamically, we can get the flow similarity value in advance. Therefore, combined with [8,9], this paper proposes an improved similarity method, which uses the probability distribution of packet size, packet length, flow rate, source port, and destination port (5- tuples) to represent benign traffic and abnormal traffic. The method can distinguish benign traffic from abnormal traffic at the ingress router. 

Recently, there have been some studies applying CNN (Convolutional Neural Network) to attack classification. CNN is better than other deep learning neural networks because it is good at extracting local features and down sampling features. The model is more suitable for processing data with more feature dimensions, such as images and text [10]. In this paper, we combine CNN and attention to extract features from the packet header and payload. The data packet header and payload features are extracted through a convolution operation, and the generated feature map is input into the attention mechanism to capture the dependence of any position of the feature map and effectively classify DDoS.

In addition, [11,12] introduced the network topology and traffic generation mode creating self-generated datasets and studied how to use attack tools to generate DDoS attacks datasets on the experimental attack and defense platform, respectively. However, the massive benign traffic is not simulated in a 5G scenario. Researchers have used many datasets [13,14,15] to evaluate the performance of their proposed intrusion detection and intrusion prevention methods. These datasets are outdated, so cannot reflect current attack types and attack methods. Therefore, collecting real datasets is a very important intrusion detection task [16].

This paper proposes a two-stage intelligent detection method for the 5G network. First, the self-generated dataset is obtained by simulating different types of attacks and 5G benign behaviors. Second, we analyze the attack behavior to select the 5-tuple features, and the similarity-based prior knowledge model is established the 5-tuple features probability distribution. This can help distinguish between benign traffic and abnormal traffic. Third, the CNN-based attention model trains abnormal traffic and optimizes the detection model. Finally, the two-stage intelligent detection model is deployed at the ingress router to achieve online detection for classifying 21 kinds of DDoS. The contribution of this article is as follows:

1. We generate a self-generated dataset that conforms to the actual traffic. The experiment simulates a large amount of benign traffic and different types of DDoS attack traffic in 5G scenarios.
2. We propose a two-stage intelligent detection model. The model includes the similarity-based prior knowledge model and the CNN-based attention model.
3. A similarity-based prior knowledge detection method for DDoS attacks is proposed. Through attack behavior analysis, we select five representative features. The probability distribution of the features is used to build a model based on the similarity with prior knowledge. The model can distinguish DDoS from benign traffic.
4. A CNN-based attention DDoS attack detection method is proposed. The CNN-based attention detection model deeply learns 29 packet payload and packet header statistical features, selects the model’s best parameters, and indicates the specific types of DDoS attacks.
5. We define a malicious detection capability metric. We apply the defined metric and common metrics to evaluate the proposed mechanism. By comparing with benchmark statistics and neural network algorithms, we show that the proposed approach performs well in terms of the detection rate, malicious detection capability, and response time.

The remainder of this paper is organized as follows. Section 2 introduces recent related work on the network intrusion detection method and dataset. Section 3 describes the proposed detection model. Section 4 presents dataset construction and evaluation metrics. Section 5 analyzes the experimental results. Section 6 concludes the paper.

## 2. Related Work

### 2.1. Dataset

A network intrusion detection system (IDS) has become an important part of the network security architecture. The IDS can analyze network traffic to identify malicious attacks. From an evaluation perspective, while datasets are important to measure the effectiveness of IDS [17], it is extremely difficult to acquire high-quality datasets. Mishra et al. [18] summarized the detection methods for the KDD99 dataset. Although the detection rate is very high, the KDD99 dataset is outdated. It may not reflect novel types of attack or the attacker methods. Divekar et al. [19] noted a high false alarm rate in the DARPA98 dataset. The NSL-KDD dataset contained transport layer attacks, but did not include low-rate and reflective amplification attacks. Kreutz et al. [20] pointed out that NSL-KDD was not a flow-based dataset and so was not suitable for flow-based intrusion detection systems. In addition, the dataset was outdated. The CAIDA DoS 2007 dataset [21] did not provide the attack events’ names and other features. The DEFCON dataset [22] only contained malicious activity. The ISCX dataset [23,24] created a profile based on the protocol, but this dataset did not provide any attack information and so it did not reflect the truth of the label. Therefore, by combining existing attack tools and attack generation methods, we deployed an experimental environment established to generate benign traffic and attack. We simulated different 5G scenarios and scripts or tools to achieve an effective and multi-types DDoS dataset.

### 2.2. Statistical Analysis Detection Method

The current research trends show that many researchers tend to use information entropy [25] and information distance [26] for statistical anomaly detection. Researchers statistically analyze traffic features to distinguish attacks from legitimate traffic [27]. 

Yu et al. [28] summarized the work on modeling malicious activities from various perspectives, discuss the pros and cons of current models. Yu et al. [29] proposed a second-order statistics-based discrimination algorithm to detect botnet attacks. However, [28,29] mainly focus on analyzing the botnet model. Yu et al. [30] used the flow correlation coefficient to measure the similarity among suspicious flows to differentiate DDoS attacks from flash crowds. However, they lack that have a fine-grained classification of DDoS. 

Callegari et al. [31] used Kullback–Leibler Divergence (KL) to evaluate the different histograms similarity to obtain the best performance. Kailath et al. [32] proposed a new measure that they have called the Bhattacharyya distance. They verified that the distance measure is easier to evaluate than the divergence. Wang et al. [8] calculated the flow score through the distribution of packet length. This method’s results demonstrate its effectiveness in detecting the botnet. However, a single feature will not stand in for all kinds of attacks. Makuvaza et al. [33] used four features to detect DDoS with high accuracy: backward packet length standard deviation, flow duration, average packet size, and flow inter-arrival time standard deviation. Beigi et al. [34] selected flow rate, duration, average packet length, and bytes per second to provide the highest detection rate. 

Harrou et al. [35] exploited the EWMA to evaluate the statistical distance, but did not verify the effectiveness of the proposed detection method. We combined the methods in [8,35] to measure flow similarity based on previous knowledge (MFPK). According to the attack behavior, we selected five features standing for the attack, and used the probability distribution of five features to represent normal traffic and attack behavior. We deployed a similarity statistics model based on previous knowledge at the ingress router, and calculated the benign traffic baseline. Then, the incoming traffic was judged as benign or abnormal. Finally, the abnormal traffic was fed into the neural network detection model. We evaluated the effectiveness of the MFPK method at detecting DDoS attacks and compared the proposed method with the existing methods [31,32]. In order to prove the effectiveness of the features selected, we compared the performance with that of existing feature selection methods [33,34].

### 2.3. Neural Network Detection Method

Neural networks are inspired by biological neurons and can also capture more complex patterns. With this method, researchers detect attacks and anomalies [36]. Convolutional networks have been widely used in natural language processing. In recent years, convolutional networks have also been used for malicious traffic detection with high accuracy. Guo et al. [37] proposed an application traffic classification algorithm based on a convolutional neural network model. The model test is carried out through the CICAndmal2017 network open dataset. Comparison with the traditional machine learning traffic classification model indicates that the convolutional neural network model is increased by 2.93% and 11.87% in accuracy and recall, respectively. However, without fine-grained classification of malicious traffic, this author only identified malicious traffic from benign traffic. The 1DCNN (1D Convolutional Neural Network) results are better performance than those of 2DCNN (2D Convolutional Neural Network) to train long sequences of traffic data [38]. The 1DCNN is suitable for processing sequence data. Wang et al. [39] proposed an end-to-end ISCX VPN-nonVPN encrypted traffic classification method with one-dimensional convolution neural networks. The experiment results on the public encrypted traffic dataset yielded significant improvements to the state-of-the-art method. However, this method is not validated in non-encrypted traffic. We chose a packet header and payload sequence features with 1DCNN in our approach. With the development of neural networks, attention mechanisms are being proposed. Jiang et al. [40] proposed four ATS models with a Sequence-to-Sequence model. An attention-based bidirectional LSTM (Long Short-Term Memory) can enhance the correlation between the generated text summary and the source text, which can prevent the spread of cumulative errors in generated text summaries. The experiments confirm that the proposed ATS model has better performance than the baseline model. This article only applied LSAT (LSTM and Attention) in natural language processing. Malik et al. [41] proposed a mechanism that combined LSCN (Long Short-Term Memory and Convolutional Neural Network) for efficient detection of multi-vector threats and attacks. The analysis showed that the proposed method outperforms other methods in terms of detection accuracy. Fu et al. [42] proposed an attention mechanism composed of a channel attention module and a spatial attention module. The channel attention module processes the different channels’ feature maps and makes the model pay more attention to those feature maps. The spatial attention module mainly processes the different feature positions on the feature map, which makes the model pay more attention to the specific feature positions. Because of the resource limitations of the hardware platform, we used the spatial attention module to capture the dependence of any two spatial features.

In our approach, we combined 1DCNN and an attention structure to classify malicious traffic for online attack detection. We used 1DCNN to learn the packet header and payload features, utilized the attention mechanism to strengthen the neural network learning capability, and further fine-tuned the identification and classification. To verify our proposed model for DDoS attacks, we compared it with the LSAT model [40] and the LSCN model [41].

In summary, firstly, we generated a self-generated dataset on the virtual platform. This dataset can stand in for traffic on the ground. Secondly, the proposed mechanism utilized similarity-based prior knowledge to distinguish benign flow from abnormal flow. Thirdly, the attention and convolutional neural network model fully learned features to detect various attacks at the ingress router.

## 3. Detection Model

In this section, we focus on introducing the two-stage detection model including a statistical detection model and a neural network detection model. 

### 3.1. Two-Stage Detection Model

**Figure 1:**
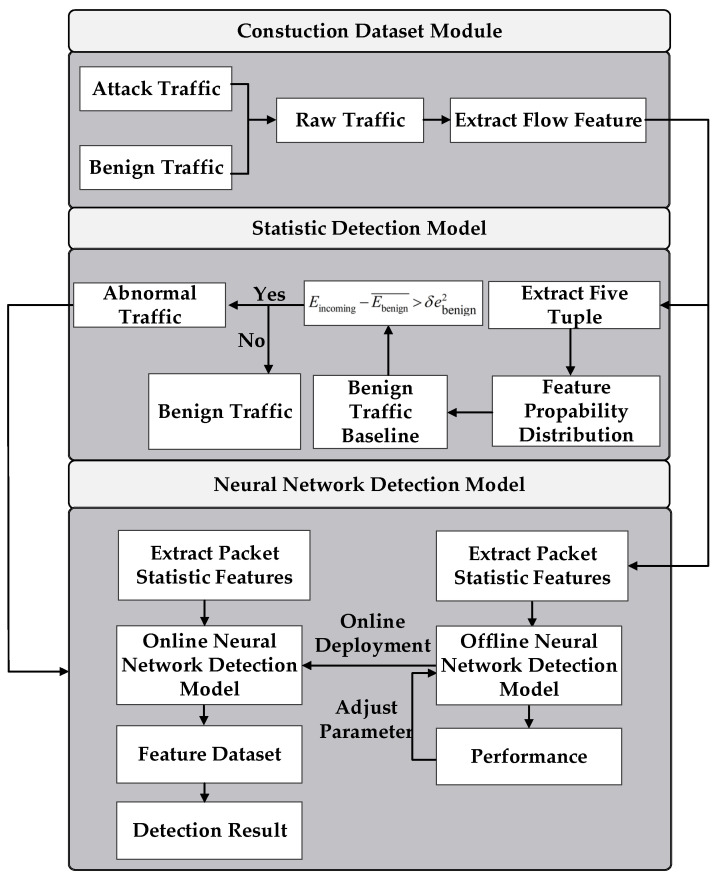
Two-stage DDoS detection model.

The two-stage detection model for detecting DDoS attacks is illustrated in Figure 1. The figure shows the steps involved in collecting network traffic data to detect attacks. The detection method consists of a construction dataset module, a statistical model, and a neural network detection model. 

In the construction dataset module, we build the prototype system and deploy attack tools. The traffic collection tool captures TCP/IP data packets, then extracts them into a flow feature dataset. The specific details about the dataset can be seen in Section 4.1.

In the statistical analysis module, we built a statistical model of benign traffic based on flow features. Based on the currently observed traffic and statistical model, we utilized statistical techniques to calculate the similarity value. If the similarity exceeds a certain threshold, the currently observed traffic is abnormal. In this paper, the abnormal events are malicious attacks.

Therefore, we regard this as a binary classification problem. In order to classify attacks and benign traffic, we select representative features by analyzing malicious behavior. 

In the botnet network, botnet hosts regularly update a library file with a server. During the update process, the packet size and length change regularly. For DRDoS, the attacker sends requests to the reflector, and the reflector sends various response packets to the victim. These response packets have the same size and length. For the application layer attack, the attackers send HTTP requests to the server over a long time to make the webserver flood; thus, the packet size and length change regularly. 

For the network layer DDoS, the attack tool can initiate flood attacks by setting the packet size and packet length. The normal user can send requests to the server and stay on the server for some time so that the user can browse the information. Usually, for benign traffic, the URLs require different packet sizes. The data packet size and length are considered traffic features that can be used to distinguish between benign traffic and attack traffic.

For low-rate attacks, it is necessary to establish a connection between the source and the destination host, and the destination host sets a minimum window to read the bytes. The transmission rate of bytes is slowly maintained by the source host and the destination host. Many useless packets increase for a short period of time in high-rate attacks (such as network layer flood, application layer flood, and reflection amplification attacks). The benign traffic rate is different from low-rate and high-rate attacks. The growth of flow numbers is very obvious. Therefore, we consider the flow rate one of the most important features.

In addition, in order to quickly achieve the effect of the attack, the attack tool specifies the victim port, and the port number also contains the attack information. Therefore, the 5-tuples include packet size, packet length, flow rate, source port, and destination port, which are used to represent malicious behaviors. 

The statistical detection module is used to establish the feature distribution of malicious behaviors and calculate the flow similarity in the time window. Then, by comparing it to the threshold, the statistical detection module outputs the abnormal traffic.

The abnormal traffic is fed into the neural network detection module in Figure 1. The detection module learns the packet headers and payload features, further classifying DDoS attacks at the ingress router. Finally, the detection module can improve the malicious traffic detection capability and classify 21 DDoS types. 

### 3.2. Statistical Detection Model

Combining the methods from [8,35], we propose a statistical detection algorithm to measure flow similarity based on previous knowledge, named MFPK. The MFPK algorithm steps are as follows:

(1) Collect flow X and Y

The traffic sampled aggregates to flows in the time interval ΔT in Figure 1. n(0≤n≤K) are feature numbers. After the malicious behavior analysis, we adopt the five features including package size, package length, flow rate, source port, and destination port. We adopt K = 5. Thus, the X and Y flow consist of five features, respectively.

(2) Compute flow X and Y similarity

The network traffic will change dynamically, which causes the flow similarity to fluctuate. We used the exponentially declining weighted moving average method to estimate the flow similarity value. This method takes into account previous data, which makes the method sensitive to traffic changes. This method can make it easier to detect attack traffic in advance.

P and U are the discrete probability distribution in the time interval ΔT. P and U are defined as follows:

(1) $$P=\left(p_{1},p_{2},\ldots ,p_{n}\right)(0\leq n\leq K)$$

(2) $$U=\left(u_{1},u_{2},\ldots ,u_{n}\right)(0\leq n\leq K)$$

The P and U probability distribution are as follows:

(3) $$P=\left(p_{1},p_{2},\ldots ,p_{n}\right)=\frac{X[a_{m}^{n}]}{M}=\left\{\frac{x[a_{m}^{1}]}{M},\frac{x[a_{m}^{2}]}{M},\frac{x[a_{m}^{n}]}{M}\ldots ,\frac{x[a_{m}^{K}]}{M}\right\}$$

(4) $$U=\left(u_{1},u_{2},\ldots ,u_{n}\right)=\frac{Y[b_{l}^{n}]}{L}=\left\{\frac{y[b_{l}^{1}]}{L},\frac{y[b_{l}^{2}]}{L},\frac{y[b_{l}^{n}]}{L}\ldots ,\frac{y[b_{l}^{K}]}{L}\right\}$$

In Equations (3) and (4), the notation of flow X is $X[a_{m}^{n}]$ and the notation of flow Y is $Y[b_{l}^{n}]$. M and L are the total number of flows in the time interval ΔT. $a_{m}^{n}$ is the *n*-th feature value in the *m*-th flow. $b_{l}^{n}$ is the *n*-th feature value in the *l*-th flow. $x[a_{m}^{n}]$ and $y[b_{l}^{n}]$, respectively, are the same *n*-th features value number. Given the positive value of the probability, we have the following relationship:

(5) $$p_{n}\gg 0,u_{n}\gg 0$$

(6) $$\sum_{n}^{K}p_{n}=1,\sum_{n}^{K}u_{n}=1$$

The similarity between P and U is given by:

(7) $$S(p_{n},u_{n})=\sum_{n=1}^{K}(\frac{\left|p_{n}-u_{n}\right|}{1+\max\left\{p_{n},u_{n}\right\}})$$

(8) $$E_{t}=\left(1-\alpha \right)S_{t-1}+\alpha S_{t}$$

In Equation (8), *t* denotes the current time window, $S_{t}$ denotes average similarity in the current time window, $S_{t-1}$ denotes average similarity in the last time window, and $E_{t}$ denotes the estimated similarity value in the current time window. α is an adjustable parameter [43]. In general, α is 0.8. In Section 5.1, we use distance to measure the traffic similarity. The distance and similarity are inversely proportional. The smaller the distance, the higher the similarity, and the larger the distance, the lower the similarity. If the probability distributions P and U are equal, the flow distance value is small. When the two probability distributions are completely different, the flow distance value will be higher. The greater the difference between the two distributions, the smaller the distance value. When the flow distance value decreases, it indicates that there may be abnormal flow in the network. In general, the normal host can always keep communicating. Thus, the normal flow has a high distance value. When an abnormal event occurs, a large number of packets exist in the network, which can decrease the distance values. The larger the distance, the less similarity between benign and attack traffic. This property can distinguish benign traffic from attack traffic according to the flow similarity.

(3) Define whether abnormal traffic exists

When we consider the network traffic dynamic, the similarity may be subject to fluctuations [44]. The threshold factor is used to adjust the detection rate; ∆*E* denotes the fluctuation of abnormal flow similarity and benign flow similarity. That is the possibility of being attacked. The ∆*E* between P and U is given by:

(9) $$\Delta E=E_{\mathrm{incoming}}-{\bar{E}}_{\mathrm{benign}}$$

(10) $$\Delta E>\delta e_{\mathrm{Benign}}^{2}$$

In Equation (10), $e_{\mathrm{Benign}}^{2}$ is the similarity variance of the benign traffic. $\delta e_{\mathrm{Benign}}^{2}$ denotes benign traffic fluctuations. When the difference between benign traffic and incoming traffic is higher than the threshold, the incoming traffic is abnormal. We discuss the similarity threshold δ in Section 5.1. The MFPK precoder algorithm is given in Algorithm 1.
**Algorithm 1** MFPK Precoder Algorithm.**Input**: Flow ***X***, Flow ***Y***, *M*, *L*, *K*;**Output**: True: abnormal, False: benign;1: **for** *m* = 0; *m* < *M*; *m* + + **do**2:  **for** *n* = 0; *n* ≤ *k*; *n* + + **do**3:  $\;x[a_{m}^{n}]\leftarrow C\mathrm{ount}(a_{m}^{n})$;4:  $\;P_{n}\leftarrow x[a_{m}^{n}]/M$;5:  **end for**6: **end for**7: **for** *l* = 0; *l* < *L*; *l* + + **do**8:    **for** *n* = 0; *n* ≤ *k*; *n* + + **do**9:     $y[b_{l}^{n}]\leftarrow Count(b_{l}^{n})$;10:   $U_{n}\leftarrow y[b_{l}^{n}]/L$;11:  **end for**12: **end for**13: $S_{\mathrm{incoming}},E_{\mathrm{incoming}}\leftarrow \mathrm{Calculate}Similarity(p_{n},u_{n})$14: $\mathrm{if}\;E_{\mathrm{incoming}}-\bar{E_{\mathrm{benign}}}>\delta e_{\mathrm{Benign}}^{2}$ **then**15:   **return** True;16: **end if**17: **return** False;

### 3.3. Neural Network Detection Model

We proposed a neural detection algorithm, named CNAT, combining CNN and attention structure to classify malicious traffic for online attack detection. However, we took the online detection time requirements into account. Thus, this article only uses the spatial attention mechanism to learn features and classify multiple attacks.

**Figure 2:**
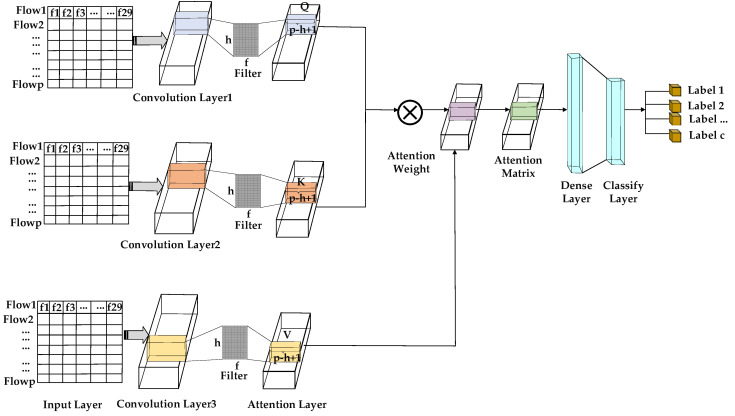
CNN-attention model.

Figure 2 shows the CNN-based attention model. The function of every layer is as follows:

Input Layer: We extracted the features of the packet header and payload in the abnormal traffic obtained by the MFPK detection method. In this first layer, it takes as input a traffic flow represented by a feature matrix F of size p∗f. F contains individual p flow vectors in the interval window ∆T, and each flow vector f is 29.

CNN layer: We used 1DCNN to train the flow features. We input these features into the feature detection suitable for processing packets’ statistical features. Each input feature matrix F was operated on by a single convolutional layer with e filters of size h∗f, where *h* is the height of each filter, and *f* is 29. Each filter covered F with a step of s to extract and learn the packet statistical features, which contains useful information for classifying DDoS. Then, each of the e filters generated a feature map of size p−h+1. We utilize the activation function to minimize the error between the true label and the predicted label. The ReLU and sigmoid are common activation functions. We introduced the ReLU activation function since ReLU is proven to be faster to train than standard sigmoid units [45], so we use the rectified linear function ReLU(x)=max{0, x}.

Attention layer: The attention mechanism can encode the sequence packet header and payload feature, and assign a weight to each feature determined by the feature similarity between the corresponding two locations.

In Figure 2, after convolutional layers, the attention layer includes query Q, key K, and value V. Q, K, and V are defined as follows:

(11) $$Q=W^{q}F;K=W^{k}F;V=W^{v}F$$

In Equation (11), the $W^{q}$ is the query matrix, $W^{k}$ is the key matrix, and $W^{v}$ is the value matrix. These three matrices are learned through a neural network. Based on Equation (11), *Q*, *K*, and *V* are generated after convolutional layers.

The similarity $S_{ji}$ between features is calculated by *Q* and *K*. The similarity $S_{ji}$ is the attention weight. The value V is operated by each attention weight with a weighted sum method. The similarity $S_{ji}$ is as follows:

(12) $$S_{ji}=\frac{\exp(Q_{i}\cdot K_{j})}{\overset{f}{\underset{i=1}{\sum }}\;\exp(Q_{i}\cdot K_{j})}$$

In Equation (12), $Q_{i}$ and $K_{j}$ are the encode with the *i*-th feature and the *j*-th feature, respectively. $S_{ji}$ is the matching degree between the *j*-th feature and the *i*-th feature.

(13) $$C_{j}=\sum_{j=1}^{f}S_{\mathrm{ji}}V_{j}$$

In Equation (13), $C_{j}$ is the features matrix aggregated by the attention module; f is the feature number; and $V_{j}$ is the encoder for the j-th feature. The attention module can improve any two feature positions similarly. Finally, the features matrix $C_{j}$ aggregates by the attention module.

Dense layer: $C_{j}$ is an input to the dense layer. We used the optimization function to minimize the loss between the real value and the predicted value. The optimization function and learning rate affect the model performance. An appropriate learning rate can reduce the error during back propagation. During back propagation, weight and bias are important parameters. Weight is the real value that is associated with each feature. Bias is to better fit the data. The model can continuously update the convolutional weight and bias. This also means that the model fully learns the relationship between packet statistical features and the label, which reduces the network complexity. $C_{j}$ is flattened to produce the final one-dimensional feature vector v. The feature vector v will be input into the classification layer.

Classification layer: v is input to a fully connected layer, and the output layer number is the abnormal types. We use the softmax function to classify the different abnormal types. In Figure 2, c is the abnormal fine-grained category label. In this article, for DRDoS, we cite the six categories in the references [46]. For botnets, application layers, network layers, and low-rate, we chose attacks that attack the effect well in each category. Thus, the botnet has four categories. The application DDoS has four categories. The network DDoS has three categories. The LDDoS has four categories. The specific traffic type can be seen in Section 4.1.

In this paper, we input standardized data by small batch. This method normalizes the input data, makes the data normalized through discarding, scaling, and translation, and prevents slow convergence or invalid features. However, the batch size is an adjustable parameter. A larger batch size does not mean better model detection performance. The larger the batch size, the more likely gradient explosion is to occur. In this situation, the neural network has no nonlinear ability, and the convolution layer does not learn local features, resulting in model overfitting. With a smaller batch size number, the convolutional layer is not enough to learn the relationship between features and labels, resulting in model underfitting. Therefore, we set the convolutional layer number to three and the filter number to one. The learning rate, batch size, and optimization function are adjustable parameters. Furthermore, considering the complexity of the model, we set the model iteration number R to 10.

1DCNN with an attention mechanism has three advantages. Firstly, the standard neural network is fully connected, and the weights of each filter in the convolutional layer are shared. Compared with the fully connected network, 1DCNN needs fewer weights and the model is lighter, which can effectively reduce the computational complexity of the model. Secondly, in the training process, 1DCNN can automatically learn features with weight w and bias b without relying on expert knowledge and time-consuming feature engineering. Finally, the attention mechanism can further capture the dependence between the packet payload and the packet header statistical features learned by the convolutional layer. For the specific features’ locations, the features are updated by aggregating the features at all locations, which focus on various features; this makes the model pay more attention to the corresponding areas of the feature map. It can enhance the detection ability of the neural network. The CNAT precoder Algorithm 2 is as follows:
**Algorithm 2** CNAT precoder Algorithm.**Input**: The feature matrix **F**; the filter e; the feature number **f**; the maximum number of iterations **R**; the convolution filter weight **w**; the convolution filter bias **b**; the query matrix ***W*^q^**; the key matrix ***W*^k^**; the key matrix ***W*^v^**;**Output**: The traffic type;1: **for** epoches = 1 to *E* **do**2:  initial w to be 0; b to be 0;3:  **for** r = 1 to **R do**4:   $z^{\left[r\right]}=w^{\left[r\right]}f+b^{\left[r\right]};$5:   $F^{\left[r\right]}=\mathrm{Relu}(z^{\left[r\right]}{+b}^{\left[r\right]});$6:  **end for**7:  $Q=W^{q}F;K=W^{k}F;V=W^{v}F$8:  $S_{ji}=\frac{\exp(Q_{i}\cdot K_{j})}{\sum_{i=1}^{f}\exp(Q_{i}\cdot K_{j})}$;9:  $C_{j}=\sum_{j=1}^{f}s_{ji}V_{j}$;10: **end for**11: Add dense layer, classification layer12: **return** The traffic label;

## 4. Methodology

In this section, we introduce how the dataset is generated, propose a new detection metric (namely, malicious traffic detection capability), and use common metrics to evaluate the two-stage detection model proposed in Section 3.

### 4.1. Dataset

The experimental platform is built based on Vmware vSphere software (http://www.vmware.com/cn.html (accessed on 9 January 2021)). The specific configuration of the virtual machine is shown in Table 1. 

**Figure 3:**
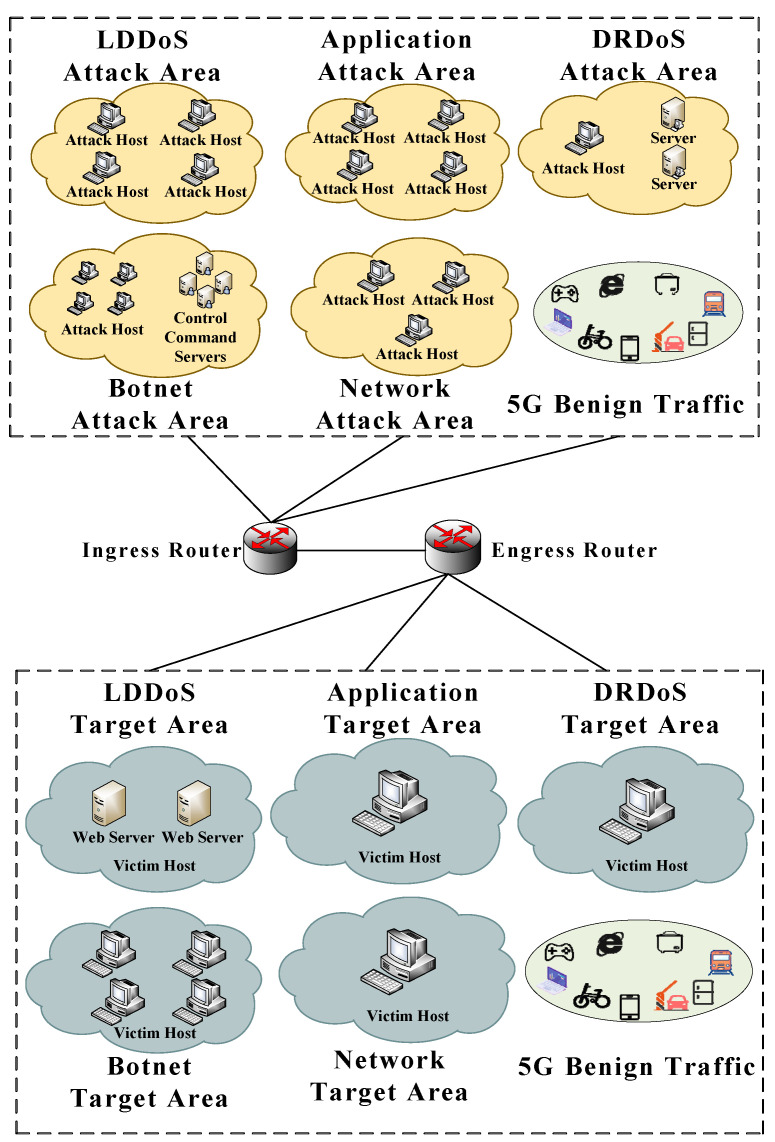
Network topology diagram.

The software environment is the Ubuntu18.04 Server system, the number of virtual cores is 8, and the memory is 16 GB. The topological structure adopted is shown in Figure 3. We simulated multiple types of botnets, application layers, network layers, low-rate, DRDoS attacks, and benign traffic of multiple 5G scenarios in the virtual machine.

The attack domain deploys the attack tools and scripts in Figure 3. After the attackers execute the tools or scripts, malicious and normal behaviors pass the ingress router to reach the target domain. The following five types of attacks are involved in the experimental platform:

Low-rate DDoS: In the LDDoS area, we used eight hosts, including two routers, four attack hosts, and two web servers. The attack hosts used Slow HTTPTest (https://github.com/shekyan/slowhttptest/ (accessed on 11 March 2021)) to send LDDoS attacks to the web server. The LDDoS attacks included slow headers, slow body, shrew, and slow read.

Application Layer DDoS: we utilized four hosts to generate four application layer DDoS. We separately used the Hulk (https://dl.packetstormsecurity.net/DoS/hulk.zip (accessed on 5 February 2021)), Webbench (http://www.ha97.com/4623.html (accessed on 5 February 2021)), and Golden Eye tools (https://github.com/jseidl/GoldenEye (accessed on 5 February 2021)) to simulate HTTP Flood attack, CC (Challenge Collapsar) attack, HTTP Post attack, and HTTP Get attack.

DRDoS: In the reflection amplification attack domain, we used one attack host and two servers. The attacker used the Scapy library (https://scapy.net (accessed on 18 February 2021)) to send fake requests to the two servers to generate DRDoS attack traffic, including Memcached, TFTP (Trivial File Transfer Protocol), Chargen, NTP (Network Time Protocol), SNMP (Simple Network Management Protocol), and SSDP (Simple Service Discovery Protocol). The victim host receives a large number of responses from the server. 

Botnet attack: In the zombie attack domain, we used four hosts and four control command servers. The control and command server are used to maintain the connection with the zombie host. According to the commands of the control command server, the zombie host will execute malicious behaviors, including Ares (https://github.com/sweetsoftware/Ares (accessed on 5 February 2021)), BYOB (https://github.com/malwaredllc/byob (accessed on 10 February 2021)) Zeus (https://github.com/Visgean/Zeus (accessed on 20 February 2021)), and Mirai (https://github.com/jgamblin/Mirai-Source-Code (accessed on 7 April 2021)).

Network layer DDoS: We used three attack hosts in the network layer attack domain. With the hping tool (https://github.com/antirez/hping (accessed on 13 March 2021)), a large number of data packets were sent to the host in the target domain. The network layer attacks included SYN, ACK, and UDP.

Benign traffic: We use the socket package (https://docs.python.org/3/library/socket.html (accessed on 15 April 2021)) to establish a connection between the client and the server to send packets. According to [47], the virtual machines simulated the 5G environment, including public services, smart homes, PC Internet, and MTC communication. This scenario generated normal communication traffic. In addition, to enrich the normal traffic category, we adopted the benign traffic in the CICIDS2017 dataset [48] as the background traffic, which can add to the traffic complexity. 

At the ingress router, we used the Tcpdump (https://www.tcpdump.org (accessed on 18 April 2021)) tool to capture the network traffic to be detected within a certain period of time. This obtained the original dataset, containing attack traffic and benign traffic.

The investigation showed that different flow features are helpful for traffic analysis, which can provide relevant communication information [49]. Several approaches utilize these features to improve the abnormal detection performance [50,51]. The information of IP flow is more comprehensive; thus, we used the CICFlowmeter tool [52] to generate flow features from the PCAP file. A network flow consists of all packets sharing a common combination of source IP, destination IP, port, and transport protocol. We extracted the network traffic with the CICFlowmeter tool, which is composed of 84 traffic flow features. The main features are the forward and backward data packet number, the data packet arrival time, the length of the data packet, the header flag count and header feature, and their statistical features, such as the minimum, maximum, average, and standard deviation. For a complete description, readers are referred to the CICFlowmeter document [52].

Our self-generated dataset contains multiple attacks and benign traffic, which are suitable for processing a large volume of traffic with neural networks. Based on the above implementation methods, Table 2 summarizes the initiation time of attack traffic and benign traffic, as well as the source and destination IP addresses. According to the timetable, we launched different types of attacks, including botnet, low-rate, network layer, application layer DDoS attacks, and DRDoS attacks. The dataset is publicly available at https://github.com/liliMpro/source_dataset (accessed on 22 February 2022).

We obtained the network traffic pcap file at the ingress router, and used the CICFlowmeter tool to extract the flow feature information of the traffic to obtain the multi-type DDoS attack dataset. Table 3 shows the flow type, number of data samples, and ratio. 

### 4.2. Evaluation Metric

The statistical analysis detection method distinguishes attack traffic from benign traffic. We utilized the detection rate to evaluate the detection performance, and used the response time to evaluate the detection period of the statistical algorithm proposed in Section 3.2.

(14) $$D\mathrm{etection}R\mathrm{ate}=\frac{D\mathrm{etectAbnormalTrafficNumber}}{T\mathrm{otalTrafficNumber}}$$

In Equation (14), the numerator is the incoming traffic that is classified as abnormal traffic through the MFPK algorithm, and the denominator is the incoming traffic.

In order to measure the neural network detection performance, we provide the following performance metrics: response time, accuracy, precision, recall, confusion matrix, and loss. The model output was used to indicate the fine-grained category of DDoS attacks. 

We utilized a Python script to save on the response time. The response time reflects the model’s complexity. The more complex the model, the longer the response time.

We base our definition of accuracy, precision, and recall on the following four previous definitions: (1) false positive (FP) is the number of benign samples that are misclassified as attack traffic; (2) false negative (FN) is the number of attack samples that are misclassified as benign traffic; (3) true positive (TP) is the number of attack samples that are correctly classified as attack traffic; and (4) true negative (TN) is the number of benign samples that are correctly classified as benign traffic [53].

Considering these previous definitions, accuracy (*A*cc) refers to the proportion of samples correctly classified in the total.

(15) $$A\mathrm{cc}=\frac{TP+TN}{TP+TN+FP+FN}$$

Precision (*P*re) indicates the proportion of correctly predicted attack traffic to the predicted attack traffic.

(16) $$P\mathrm{re}=\frac{TP}{TP+FN}$$

Recall (*R*ec) indicates the proportion of correctly predicted attack traffic to all attack traffic samples.

(17) $$R\mathrm{ec}=\frac{FN}{TP+FN}$$

The normalized confusion matrix is mainly used to analyze the classification results of the detection model and the matching degree between prediction type and actual type.

The loss function is used to evaluate the difference between a single traffic prediction type and the real type. The smaller the loss function, the better the robustness of the detection model. The loss function is mainly in the model training stage. After the batch size data are fed into the model, the forward propagation output is the predicted value, then, the loss function calculates the loss value. According to the loss value, the model updates each parameter to reduce the loss through back propagation, and to make the predicted value generate closer to the real value and achieve the learning purpose. Therefore, the purpose of the loss function is to reduce the loss of each iteration. This is a multi-classification problem, and we use the sparse categorical cross-entropy loss function as our loss function:

(18) $$\psi =-\frac{1}{N}\sum_{m\in M}\sum_{s\in S}1_{m\in M}\log p(m\in s)$$

where ψ is the sparse categorical cross-entropy loss function, m is the sample number, and s is the class label. 

In addition, in order to analyze the online detection ability, we defined a new evaluation metric: malicious traffic detection capability. Malicious traffic detection capability (MTDC) means the ability to detect attack traffic after online detection. The equation is as follows:

(19) $$\mathrm{MTDC}=\frac{1}{\sum_{i=1}^{k}T_{i}/\sum_{i=1}^{k}A_{i}}$$

where MTDC is the malicious traffic detection capability, *A_i_* represents the total traffic of attack traffic type i, k indicates the type of attack traffic, and *T_i_* indicates the traffic correctly detected as attack traffic type *i*.

## 5. Experimental Results

In this section, we verify the detection performance of the two algorithms proposed in Section 3 and compare them with benchmark algorithms. Using the MFPK algorithm, we select the time window ΔT and the threshold δ parameters that affect the detection performance. The time window selected was the 90 s, and the threshold δ was 1.1. Compared with the existing methods [31,32,33,34], the MFPK can have a higher detection rate and lower response time. 

For the neural network model, we first selected the CANT neural network model hyperparameters. Through our experimental analysis, we determined that the batch size was 15, the optimizer Adam, and the learning rate was 0.0001. After setting these hyperparameters, we trained the CNAT model proposed in the offline phase, deployed the trained model at the ingress router, and utilized the CNAT model to detect abnormal traffic in the online phase. Compared with the existing model [40,41], the CNAT model has a higher malicious traffic detection capability and a shorter response time. The experimental results show that the model can also detect 21 different types of DDoS attacks at the ingress router.

### 5.1. Distinguishing Attack Traffic from Benign Traffic

We used a statistical analysis detection method to distinguish attack traffic from benign traffic. In the statistical detection model, the time window ∆T of the MFPK method and threshold δ are two important parameters. We collect network traffic periodically at the ingress router, and the time window will be a key feature that affects the detection result. If the time window is too small, some features may take a long time to capture and it may not be possible to capture all the traffic. If the time window is too large, this will take a long time to detect. Therefore, the time window ∆T affects the performance of the statistical model. If δ is too high, many DDoS packets will be judged as normal packets, which will decrease the similarity between normal and DDoS packets. If δ is too small, many normal packets will be deemed DDoS attacks. To obtain the best parameters for ∆T and δ, we performed experiments and analyses.

#### 5.1.1. Similarity vs. Time Window

**Figure 4:**
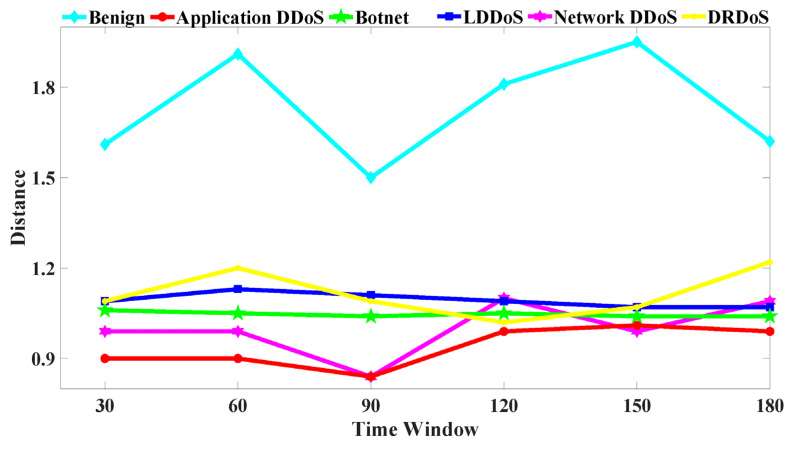
Distance of MFPK method between benign vs. attack traffic.

The reason for using distance to represent similarity has been explained in Section 3.2. As shown in Figure 4, the *y*-axis represents the difference of the distance values, and the *x*-axis represents the time window used for computing the distance values.

In the case of attack traffic, the attackers generate small packets in a short period of time, and the attack traffic is more similar to legitimate traffic. This causes significant deviation from the legitimate traffic. In different time windows, the similarity of benign traffic is lower than that of attack traffic. Benign traffic is composed of 5G legal requests and human interaction behaviors. We simulated different 5G application scenarios (such as smart home, public service, and MTC communication). The request packet size and request period are different in each scenario. In the CICIDS dataset, benign traffic is generated by human interaction (e.g., HTTP, HTTPS, SSH, or email). To access the website, the user sends a normal request. During the request generation process, a data packet is randomly generated. When a network resource is requested, the user will stay on the website for a period of time, and the user can browse the web. Thus, the number of the flow is small in the process of requesting resources. Combining both situations, the data packets are random, large packets are transmitted, and the payload of the packets is various in the normal flow, so the similarity of the normal flow is low.

In Figure 4, the attack traffic distance is generally smaller than the benign traffic, so the attack traffic similarity is high. The distance of benign traffic increases slowly from 30 s to 60 s. The user starts to send different request packets to the server, causing the distance value to increase, as well as the similarity to become smaller. From 60 s to 90 s, the user establishes a connection with the server for a certain period of time, and the user stably sends the same packet size to the server to transfer files. Therefore, the benign traffic distance value decreases, and the similarity is greater. When the user requests resources, the user will stay on the website for a period of time to browse the website resources. At this time, there are fewer packets in the network, so the distance value increases from 90 s to 150 s. From 150 s to 180 s, when the user finishes browsing the website, the user will request that the server close the connection, and the server will send response packets to the client. Therefore, the benign traffic distance value drops, and the similarity becomes greater.

For the botnet, the botnet hosts need to inquire about the server status within a certain period of time. Then, the botnet hosts download the corresponding binary configuration file, so as to that maintain the communication between the server and the botnet; however, there are few packets in the communication traffic. Thus, the botnet traffic changes are stable and the similarity changes smoothly under different time windows.

For the LDDoS, the attackers send a burst to the victim host. As time goes on, there are incomplete or short packets in the network, which leads to the distance increasing and then gradually stabilizing. However, there are fluctuations in the network traffic, which means that the LDDoS distance value will be affected by the network conditions.

For application layer attacks, the application attackers send frequency HTTP requests to the server, which will affect the attack packets similarity. From 60 s to 90 s, the reason for the distance decrease is that the attacker sends a few HTTP request packets. From 90 s to 150 s, although the attacker continues to send HTTP request packets to the server, the network traffic fluctuation will cause noise, and the noise will affect the similarity. Thus, the distance value will increase during this period, and the similarity will decrease. Both network layer and application layer attacks are flood attacks. For network layer DDoS, attack tools specify the length of the data packet, the number of data packets per second, and the port number of the target host. These parameters will make the distribution of flow features consistent. The similarity will increase over time.

The DRDoS attacker sends forged requests to the server, and the victim receives responses from the server. As time goes on, the response packets number gradually increases, so the distance value decreases and the similarity become greater. However, from 120 s to 180 s, the background traffic can disturb the distance value in the experimental platform, so the distance value increases. In general, the DRDoS distance value is lower than that of benign traffic, and the similarity is higher than that of benign traffic. 

In summary, we choose the time window 90 s. This results in an apparent deviation of the similarity value between attack traffic and benign traffic in the shortest time.

#### 5.1.2. Detection Rate vs. Threshold Value

**Figure 5:**
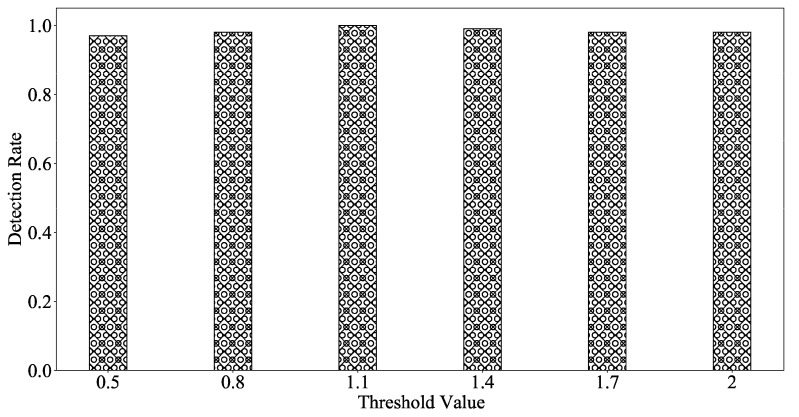
Detection rate of the MFPK method.

As can be seen from Figure 5, the size of the threshold δ will affect the detection rate. The threshold δ is not proportional to the detection rate. If the threshold δ is too small, the attack traffic will be misjudged as benign traffic. If the threshold δ is too high, the benign traffic will be misjudged as attack traffic. Due to the network traffic variability, we have done a large number of experiments, and the value of the threshold δ ultimately ranges from 0.5 to 2 in this section. As shown in Figure 5, when the thresholds δ range from 0.5 to 2, the detection rate is close to 1. In Figure 5, when the threshold value is 1.1, the detection rate reaches 1.

In summary, we choose a threshold value δ of 1.1 because it can clearly distinguish between abnormal and benign traffic and the detection rate reaches 1.

#### 5.1.3. Comparison with Other Methods

Callegari et al. [31] pointed out that KL can measure the difference between two probability distributions, so this is usually used to detect anomalies. Kailath et al. [32] proposed the Bhattacharyya distance which can measure the similarity of two discrete probability distributions and the authors pointed out that the Bhattacharyya distance has found several applications in classical statistics. Therefore, KL and Bhattacharyya are commonly used to measure similarity. In this article, we compare the proposed method with the KL and Bhattacharyya distance methods to verify the effectiveness of our method. 

**Figure 6:**
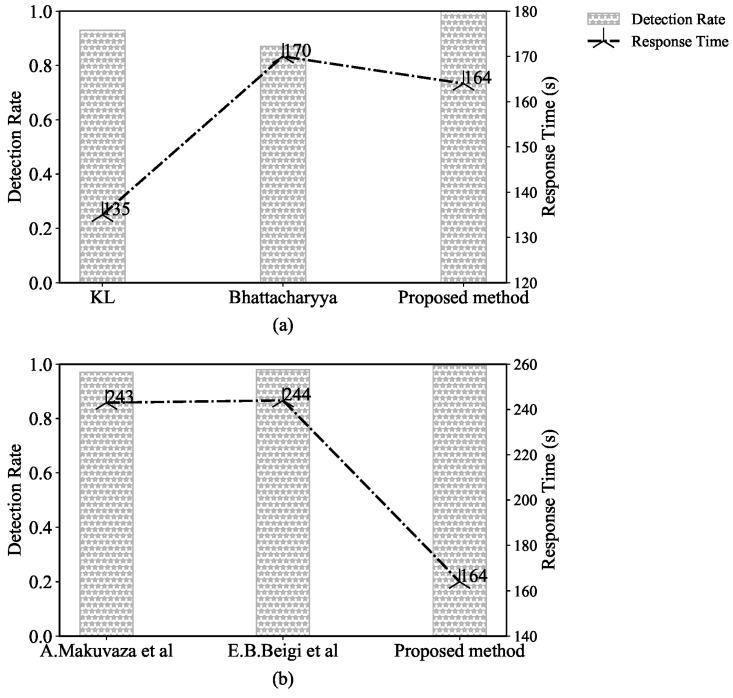
Detection rate of popular similarity methods: (**a**) Contrasting statistical methods; (**b**) contrasting feature selection methods.

In Figure 6a, the horizontal axis gives different similarity methods, the left vertical axis is the detection rate of different methods, and the right vertical axis is the response time. It can be seen from the figure that the similarity method proposed has the highest detection rate, which effectively measures the similarity and completely distinguishes between benign flow and abnormal flow. The KL and Bhattacharyya algorithms can achieve 0.96 and 0.90 detection rates, respectively. This indicates that the similarity between benign traffic and abnormal traffic is high, which indicates that some abnormal traffic cannot be distinguished by the threshold. Finally, both the KL and Bhattacharyya algorithms can misjudge abnormal flow as benign flow. 

Figure 6a shows that the detection time of the KL algorithm is at least 135 s, the proposed method’s is 164 s, and the Bhattacharyya algorithm’s is 170 s. All three algorithms have a similar response time, which is related to the algorithms’ complexity and the traffic flows. If the simulated attacks are high-speed attacks (such as application layer attacks and network layer attacks), the attack packets generated by these attacks are sudden. Thousands of flows may be collected within a time window of 90 s, which results in a long response time. If a low-rate attack (such as LDDoS) is simulated, the packet transmission rate is similar to that of benign traffic, so the traffic collected in the time window may number in the hundreds, resulting in a short response time. In sum, the proposed method can achieve both a high detection rate and an appropriate response time.

In Section 3.1, we analyzed and selected features that can represent the attack behavior. To prove the effectiveness of the features selected, we compared the performance with the existing feature selection methods [33,34]. 

In Figure 6b, the horizontal axis is different feature selection methods, the left vertical axis is the detection rate of different methods, and the right vertical axis is the response time. It can be seen that the proposed feature is the highest and the response time is the shortest. Both [33,34] used the flow duration time. In DDoS attacks, there is usually sudden traffic, which leads to an increase in the number of small packets. Therefore, the duration of each flow is short. In benign traffic, there are high bandwidth and large file transmission, which leads to a decreased flow number. Thus, the flow duration time is long [54]. Although the flow duration can distinguish between attack traffic and benign traffic, the network traffic is unstable, and the attack flow duration is not concentrated in a specific value. When calculating the similarity algorithm, it is necessary to increase the response time. Therefore, although both methods have a high detection rate, they are not suitable for online detection.

In summary, according to our experimental comparison and analysis, the proposed method has a high detection rate and a low response time.

### 5.2. Offline Train

This section mainly introduces the offline training module, which includes CNAT model tuning, and verifies the model detection ability. 

In the offline phase, the experiment has been implemented in Python v3.7 (https://www.python.org/downloads/release/python-373/ (accessed on 3 December 2021)) using Keras API v2.2.4 (https://github.com/keras-team/keras (accessed on 3 December 2021)). We used TensorFlow2.1 (http://tensorflow.google.cn/install ((accessed on 3 December 2021)) with GPU support enabled by cuDNN (https://docs.nvidia.com/deeplearning/sdk/pdf/cuDNN (accessed on 3 December 2021)) a GPU-accelerated library for deep neural networks.

After applying the statistical detection module in Figure 1, we cleaned the abnormal dataset, including deleting outliers and so on. The abnormal dataset is composed of 84 traffic flow features with the CICFlowmeter tool. Then, we selected 29 packet payload and header effective features from the abnormal dataset in Table 4. Then, we divided them into a training set and a testing set at a ratio of 7:3. The dataset is shown in Table 5.

Optimizing the hyper-parameters is a necessary step to maximize the model accuracy and minimize the model loss. They influence the training process and the model complexity. By comparing different parameters, we can obtain the CNAT model with the best performance and save the relevant model structure and parameters. This section adjusts the three parameters described in Section 3.3, namely the batch size, optimizer, and learning rate. Based on our experiments, we choose the hyper-parameters’ values based on the tuning results.

#### 5.2.1. Performance vs. Batch Size

**Figure 7:**
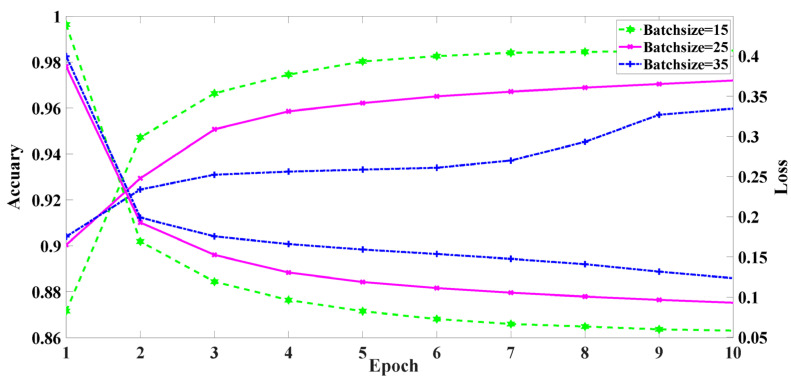
The model performance of different batch size.

Figure 7 shows the impact of batch size on model accuracy and loss. Batch size is the number of samples selected for one epoch of training. Each color represents a different batch sample size. The *x*-axis is the epoch; the *y*-axis is the accuracy rate and the loss.

As can be seen from Figure 7, with the increase in batch size, the training accuracy slowly increases and the loss slowly decreases. When the batch size is 15, the 10 epochs’ loss is 0.05 and the accuracy is 0.98. When the batch size is 25, the minimum loss is 0.09 and the accuracy is 0.97. When the batch size is 35, the loss decreases from 0.39 to 0.12, and the accuracy increases from 0.90 to 0.95. The experimental results show that a small batch size tends to converge to flat minimization, which only changes slightly in the small neighborhood of minimization. Flat minimization is easier to converge, and can quickly find the direction of loss function decline, while large batch training converges to sharp minimization, which causes the loss to fluctuate greatly. Therefore, the training model with a small batch has better generalization performance and increases the model’s robustness. The batch size selected for this paper is 15.

#### 5.2.2. Performance vs. Optimizer

**Figure 8:**
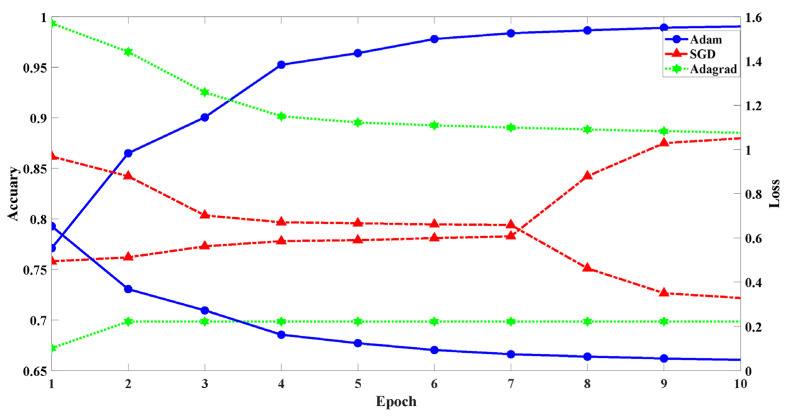
The model performance of different optimizers.

To determine the most suitable optimizer for efficiently detecting DDoS, we analyzed the learning loss and accuracy when the Adam, SGD (Stochastic Gradient Descent), and Adagrad optimization algorithms were used to train the CNAT model. Figure 8 shows the impact of different epoch times on the model accuracy and loss.

Each color represents a different kind of optimizer. The *x*-axis is the epoch; the *y*-axis is the accuracy rate and the loss. The blue line is the accuracy and loss rate of the Adam optimizer. When the number of iterations is in the interval (1, 5), the accuracy increases and the loss decreases. After the fifth epoch, the changing trend of accuracy and loss maintain a relatively stable state. The accuracy rate slowly increases and the loss slowly decreases. Finally, the loss decreases from 0.65 to 0.004 and the accuracy increases from 0.77 to 0.99, which indicates that the model is constantly fitting data.

The red line is the accuracy and loss rate of the SGD optimizer. When the number of model training iterations is in the interval (1, 3), the accuracy of the model increases from 0.75 to 0.77, and the loss decreases from 0.96 to 0.70. When the iteration number of model training is in the interval (3, 7), the accuracy and loss are less affected by the number of iterations. With the increase in the number of iterations, the accuracy increases from 0.78 to 0.87, and the loss decreases from 0.65 to 0.32. The green line is the accuracy and loss of the Adagrad optimizer. With the increase in the number of training epochs, the data are continuously transmitted to the neural network. When the number of training rounds is 10, the accuracy is only 0.69 and the loss is 1.07. The loss is the error between the real label and the predicted label. The smaller the error, the better the neural network detection performance. Based on the comprehensive consideration, this paper selects Adam as the optimizer.

#### 5.2.3. Performance vs. Learning Rate

**Figure 9:**
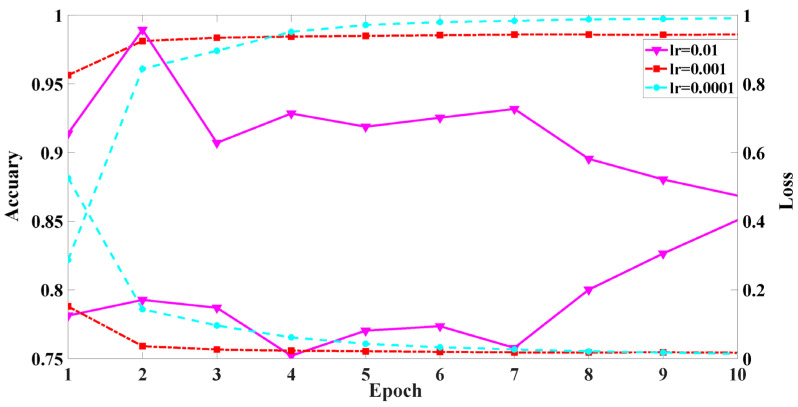
The model performance of different learning rates.

Figure 9 shows the impacts of different learning rates on the model accuracy and loss. The learning rate is one of the key parameters for training neural networks. Each color represents a learning rate. The *x*-axis is the epoch; the *y*-axis is the accuracy rate and the loss. The purple line is the accuracy rate and loss with a learning rate of 0.01. With the increase in the number of epochs, the accuracy rate and loss curve change become unstable, because the learning rate controls the range of weight parameters after updating each epoch. The higher the learning rate, the greater the weight update range. This achieved the minimum value of the loss function. The weight parameter values continue to diverge at both ends of the extremely optimal value. Therefore, with the increase in the number of epochs, the loss does not decrease and the accuracy does not increase. When training to 10 epochs, the accuracy is 0.85 and the loss is 0.47.

The red line shows the accuracy and loss with a learning rate of 0.001. It can be seen from the figure that the loss with a learning rate of 0.001 is significantly more stable than the loss curve with a learning rate of 0.01. The loss decreases from 0.15 to 0.01, and the accuracy increases from 0.95 to 0.98. However, because the learning rate decreases, the parameter update speed slows down. When the number of iterations is in the interval (1, 2), the loss decreases. However, with the increase in the number of epochs, the model loss and accuracy remain unchanged, so it spends more training resources to obtain the optimal parameter value.

The cyan line is the accuracy and loss curve with a learning rate of 0.0001. It can be seen from the figure that when the epoch number is 2, the accuracy increases from 0.82 to 0.96, and the loss rate decreases from 0.52 to 0.14. Relative to the learning rate of 0.001, a learning rate of 0.0001 is better for rapidly finding the convergence direction. When the number of epochs is 10, the accuracy rate is 0.99 and the loss rate is 0.01. Through the above analysis, we selected a learning rate of 0.0001 for this paper. According to the above parameter, the CNAT model accuracy reached 0.98.

After the parameter configuration of the CNAT model, we retrained the model, saved the trained model, and utilized it in the online detection module.

#### 5.2.4. Comparison with Other Methods

In order to verify our proposed model for DDoS attacks, we compared it with the LSAT model [40] and the CNLS model [41]. Then, the best deep learning model has been selected for the online detection of DDoS attacks. The experiment used evaluation metrics in terms of response time, precision, recall, and malicious traffic detection capability. Table 6 gives the detection results.

For the botnet multi-classification, it can be seen from Table 6 that CNAT has the highest malicious traffic detection capability. Compared with LSAT and CNLS, CNAT increased by 16 and 28, respectively. In the three methods, the Ares botnet precision and recall can reach more than 0.96. Regarding the BYOB botnet, LSAT has the highest precision, reaching 0.91. CNLS precision is 0.85 and CNAT precision is 0.81, respectively. CNAT has the highest recall, reaching 0.97. LSAT is reduced by 0.29 and CNLS is reduced by 0.27. For the Zeus botnet, LSAT has the highest precision of 0.99. CNAT has the highest recall of 0.93. In the three methods, the precision and recall of CNLS are lower than those of the other two methods, which are 0.44 and 0.09, respectively. The precision and recall of Mirai can reach more than 0.9. According to the above data analysis, the detection performance of CNAT and LSAT is the best, but the CNAT response time is the lowest. It takes 20 min to train the deep learning model. However, the response time of the other two algorithms is at least 100 min. The higher the complexity of the algorithm, the longer the neural network training time. Considering the algorithm’s complexity and limited hardware resources, CNAT has the best detection ability for botnet multi-classification.

The LSAT, CNLS, and CNAT models have similar precision and recall for each traffic type at the network layer. The precision and recall rates are both 0.99. The malicious traffic detection capabilities of the three models are 99, but the CNAT response time is the lowest.

For LDDoS attacks, the LSAT and CNLS malicious traffic detection capabilities are both 75. The malicious traffic detection capabilities of the CNAT model are 71. It can be seen from Table 6 that the LSAT model shows a large difference in the precision and recall of each traffic type. In particular, the Slow Read precision is only 0.57, and the Slow Headers precision is 0.76. The Slow Headers recall is 0.6. The precision and recall of the CNLS model for the five LDDoS are better than the LSAT model, but will produce 0.77 precision when we identify Slow Headers. Although the precision of Slow Read and the recall of Slow Headers of the CNAT model are lower than those of the other two models, the precision and recall of the CNAT model for identifying other traffic are better than those of CNLS. For Shrew attacks, the precision can reach 0.99, and the Slow Body precision can reach 0.95. The response time of the CNAT model is the shortest, at only 21 min. Therefore, the CNAT model has a lower response time and a better performance for LDDoS attacks.

For application layer attacks, the CNLS model has the highest malicious traffic detection capability, reaching 64. For the CC attack, the precision of the CNLS model is the best, and the LSAT model has the best recall. For the HTTP Flood attack, the CNAT model has the best performance in terms of precision and recall. For the HTTP Get attack, the LSAT model has the best precision, reaching 0.79. The CNAT model has the best recall, reaching 0.48. For the HTTP Post attack, the CNLS model has the best performance in terms of precision and recall. This is because HTTP Post and HTTP Get attacks both send a large number of requests packets after the TCP three-way handshake. Therefore, it takes a long time to get HTTP response packets. The two attacks types’ behavior is similar. Therefore, for HTTP Post and Get attacks, the detection performance of the three models is poor. According to the above analysis, although the performance of the CNAT model is relatively poor, the CNAT response time is the shortest. Thus, considering the performance and response time, the CNAT model is the best detection model.

For DRDoS attacks, all of the models show similar performance in terms of detection metrics, but CNAT has the shortest response time.

Based on the above analysis, it can be concluded that the CNAT model proposed is more suitable for detecting 21 DDoS attack types including botnet, LDDoS, Network, Application, and DRDoS attack.

### 5.3. Online Detection

After offline training experiments and analysis, the CNAT model showed excellent detection performance. We have further illustrated that this model’s performance is optimal for online detection. In this section, experiments compare the CNLS, LSAT, and CNAT performance in terms of malicious traffic detection capability and response time. Finally, we selected and deployed the best model at the ingress gateway to achieve a fine-grained classification of DDoS attacks.

#### 5.3.1. Comparison with Other Methods

First, we replayed the DDoS dataset described in Section 4.1, utilizing Tcpdump to capture the network traffic within the 90 s time window, as well as extracting the flow feature information through CICFlowmeter at the ingress router. Then, we utilized the similarity detection model based on prior knowledge in Section 3.2. It can distinguish between benign traffic and abnormal traffic. The abnormal traffic feeds into the trained detection model and the model outputs detection prediction labels and real labels. Eventually, we saved the model response time and the malicious traffic detection capability, and finally realized the online detection of DDoS attacks and a reduction in malicious traffic in the network.

**Figure 10:**
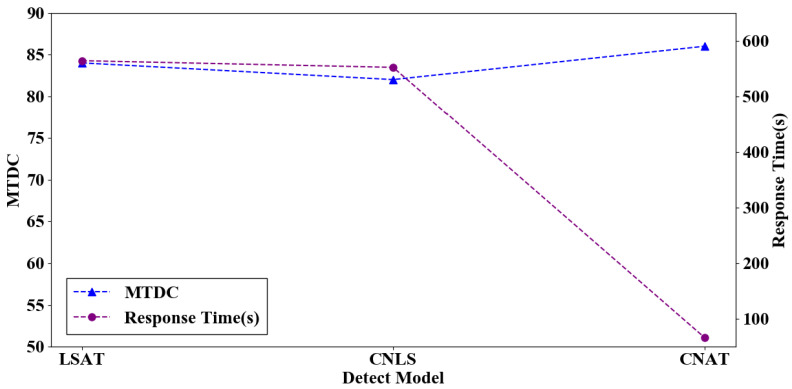
The different model performance.

In this section, the MFPK detection model is used to distinguish benign traffic from abnormal traffic; then the CNLS, LSAT, and CNAT models are compared, and the optimal detection model is selected. Figure 10 shows the malicious traffic detection capability and response time.

In the CNLS model, the convolutional neural network learns the features’ spatial information, but the features in the self-generated dataset have no temporal relationship, so the LSTM learns less temporal information from the features, which means that the model’s detection capability is poor. As can be seen from Figure 10, CNLS has the worst malicious traffic detection capability. The MTDC is only 82, and the response time is 552 s. The LSTM is suitable for processing time series features, but the packet payload and packet header features are spatial features, so LSTM cannot learn the comprehensive features information. However, the attention mechanism can analyze the relationship between features and labels, and assign appropriate weights to features. The model can focus on learning features according to the assigned weights, which can bring useful information to the model and eliminate the weakness that LSTM cannot fully learn features. Therefore, the LSAT model performance is second-best. The MTDC is 84, and the response time is 564 s.

In the CNAT model, CNN is suitable for processing sequence features. For malicious traffic detection, packet features are sequence features. The CNN learns the spatial information of packet payload and header features, and then uses attention to strengthen the model learning capability, which can improve the malicious detection capability. The model malicious detection capability is 86 and the response time is 66 s.

After the above analysis, the CNAT model proposed can use the convolutional layer to mine the packet payload and header features; furthermore, the attention mechanism can fully exploit these features’ information. This effectively distinguishes between different DDoS attack types. Therefore, the CNAT model proposed has the best malicious traffic detection capability and response time.

#### 5.3.2. Online Detection Performance

**Figure 11:**
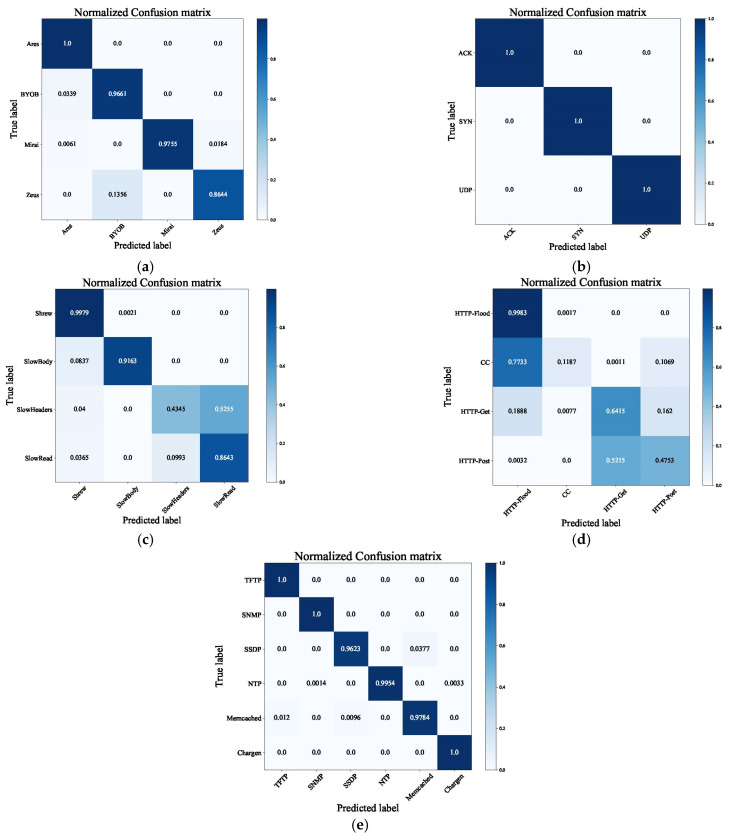
Normalized confusion matrix. (**a**) Botnet normalized confusion matrix; (**b**) Network normalized confusion matrix; (**c**) LDDoS normalized confusion matrix; (**d**) Application normalized confusion matrix; (**e**) DRDoS normalized confusion matrix.

The best CNAT model is deployed to implement online detection at the ingress router. We evaluated the CNAT model detection performance with a confusion matrix. The confusion matrix can visually represent each type of classification situation. Figure 11 shows the CNAT normalized confusion matrix performance for detecting various attacks.

Figure 11a is the botnet normalized confusion matrix, and all four botnet types are above 0.85. The Ares, BYOB, and Mirai accuracy are all above 0.95, and these attack types have less traffic misidentified as other attack types. Among them, the Zeus accuracy is poor. Zeus and BYOB will continuously download necessary malicious files from the command and control (C&C) server. Their attack behaviors are analogous, which results in Zeus being misjudged as BYOB.

Figure 11b is the network normalized confusion matrix. The ACK, SYN, and UDP attack behavior are not similar. The ACK attack means that the attack host sends a large number of ACK packets. The SYN attack means that the attack host sends a large number of SYN packets, and the UDP attack means that the attack host sends a large number of UDP packets. All three attacks are flood attacks, but the attack behaviors are different. The CNAT model can effectively learn the packet payload and header features, and the model can completely classify the three network attacks.

Figure 11c is the LDDoS normalized confusion matrix. The Shrew and Slow Body accuracy can exceed 0.9. Show Read and Slow Headers are misidentified as other attacks. The Slow Read attacker sends a request file to the attack target, and the attack target reads the response data slowly. Therefore, the attacker and the attack target occupy the HTTP connection for a long time, so it may fail to respond to normal requests and services. The Slow Headers attackers continue to send incomplete packets, which can occupy HTTP resources all the time. Therefore, both of them occupy HTTP connections for a long time to generate denial of service, and their attack behaviors are similar. The Slow Headers and Slow Read attacks will misjudge each other. The Slow Headers accuracy reaches 0.43, and the Slow Read accuracy reaches 0.86.

Figure 11d is the application normalized confusion matrix. The HTTP flood accuracy reaches 0.99. The CC and HTTP flood attackers simulate flood attacks, so a large number of CC attacks will be misjudged as HTTP flood attacks. Both HTTP Post and HTTP Get attacks set the Golden Eye tool parameters to simulate attack traffic. They are too similar and it is easy to confuse them. The HTTP Get attack and HTTP Post attack will misjudge each other. According to Figure 11d, the CNAT model has a poor performance in identifying application attacks. In the future, we will propose a detection model for application attacks that can learn application attack features and classify specific attacks with high performance.

Figure 11e is the DRDoS normalized confusion matrix. The DRDoS attacker sends a small number of forged requests, eliciting a large number of responses from the server. All DRDoS attacks use the server to reflect traffic, but different DRDoS attacks have different specific traffic amplification principles. Therefore, the CNAT model can distinguish six types of DRDoS attacks with packet features, and the accuracy is above 0.95.

In summary, we deployed the CNAT model to implement online attack detection. The model can effectively detect most DDoS types. The model malicious traffic detection capability is 86, and we reduced the malicious traffic at the ingress router.

## 6. Conclusions

For DDoS attacks caused by 5G, we proposed a two-stage intelligent detection DDoS attack mechanism that combines similarity-based prior knowledge and CNN-based attention to detect multiple types of DDoS attacks. We simulated a variety of attacks and benign traffic in the virtual platform, constructed a DDoS dataset, and applied the detection method to this dataset. The experiments showed that the similarity detection method can distinguish between benign and abnormal traffic. The CNN-based attention model deeply learns packet payload and header statistical features. It can effectively detect multiple types of DDoS attacks. Furthermore, we defined a metric including malicious detection capability. Compared with existing statistical and neural network methods, the proposed model has the shortest response time, the highest detection rate, and an adequate malicious traffic detection capability.

However, the detection performance for specific types is insufficient. In the future, the following four research directions are worth exploring:

1. We will select representative features for each attack type, establish the neural network model suitable for this attack type, and improve the multi-classification performance by optimizing parameters.
2. To verify the effectiveness of the generated self-generated dataset, we must further verify the effectiveness of the detection method on the existing benchmark dataset.
3. We will introduce research on the interpretability of the deep learning model, and visualize the neural network training process. Furthermore, we will analyze the relationship between model weights and detection results.
4. We will analyze the attention layer parameters and further adjust and optimize the model structure, which can enhance the model’s detection capability.

## Figures and Tables

**Table 1:** Virtual machine configuration.

Configure	Type
System	Ubuntu 18.04 live server
Hard Disk	32 G
RAM	16 GB

**Table 2:** DDoS datasets timetable.

Collection Time	SrcIP	DstIP	Label
11 May 2021, 20.00–23.50 13 May 2021, 15:00 19 May 2021, 12:00	10.1.0.20–10.1.0.30	10.1.1.1	Benign
	10.1.0.1	10.1.1.1	Ares
	10.1.0.2	10.1.1.2	BYOB
	10.1.0.4	10.1.1.4	Mirai
	10.1.0.7	10.1.1.99	Zeus
22 May 2021, 15:31–15:56	12.1.0.1	12.1.1.1	CC
	12.1.0.2	12.1.1.1	HTTP-Flood
	12.1.0.3	12.1.1.1	HTTP-Post
	12.1.0.4	12.1.1.1	HTTP-Get
	12.1.1.20–12.1.1.30	12.1.0.3	Benign
22 May 2021, 20:14–20.25	12.1.0.4	12.1.0.3	Memcached, Chargen, NTP, SSDP, SNMP, TFTP
	12.1.1.20–12.1.1.30	12.1.0.3	Benign
23 May 2021, 11:08–11:11	13.1.0.3	13.1.1.1	SYN
	13.1.0.20–13.1.0.30	13.1.1.1	Benign
23 May 2021, 11:13–11:16	13.1.0.2	13.1.1.1	ACK
	13.1.0.20–13.1.0.30	13.1.1.1	Benign
23 May 2021, 11:18–11:21	13.1.0.1	13.1.1.1	UDP
	13.1.0.20–13.1.0.30	13.1.1.1	Benign
23 May 2021, 15:35–16:15	11.1.0.1	11.1.1.1	Slow Headers
	11.1.0.2	11.1.1.1	Slow Body
	11.1.0.3	11.1.1.1	Slow Read
	11.1.0.4	11.1.1.1	Shrew
	11.1.0.20–11.1.0.30	11.1.1.1	Benign

**Table 3:** Traffic type and number.

Type		Proportion	Number
Benign	Benign	0.3127	880,693
Network DDoS	ACK	0.0465	131,072
	UDP	0.0464	130,844
	SYN	0.0448	126,415
LDDoS	SlowBody	0.0348	98,148
	Shrew	0.0157	44,280
	SlowHeaders	0.0342	96,542
	SlowRead	0.023	64,997
Botnet	Ares	0.252	709,748
	BYOB	0.001	2926
	Mirai	0.0007	2251
	Zeus	0.0001	327
DRDoS	TFTP	0.0141	39,977
	Memcached	0.0137	38,586
	SSDP	0.0044	12,513
	NTP	0.0033	9319
	Chargen	0.0004	1269
	SNMP	0.0002	582
Application DDoS	CC	0.09	253,525
	HTTP-Get	0.0225	3435
	HTTP-Flood	0.0202	56,886
	HTTP-Post	0.0183	51,556

**Table 4:** Features subset.

No.	Feature Name	No.	Feature Name
1	Total Fwd Packet	16	Subflow Bwd Bytes
2	Total Length of Fwd Packet	17	Total Bwd packets
3	Fwd Packet Length Max	18	Total Length of Bwd Packet
4	Fwd Packet Length Min	19	Bwd Packet Length Max
5	Fwd Header Length	20	Bwd Packet Length Min
6	Fwd Packet Length Mean	21	Bwd Header Length
7	Fwd Packet Length Std	22	Bwd Packet Length Mean
8	Fwd Segment Size Avg	23	Bwd Packet Length Std
9	Packet Length Min	24	Bwd Segment Size Avg
10	Packet Length Max	25	Packet Length Variance
11	Packet Length Mean	26	Average Packet Size
12	Packet Length Std	27	Fwd Segment Size Avg
13	Subflow Fwd Packets	28	Bwd Segment Size Avg
14	Fwd Seg Size Min	29	Subflow Bwd Packets
15	Subflow Fwd Bytes		

**Table 5:** Abnormal dataset.

Type	Number of Abnormal Traffic Events
Training Set	1,607,313
Testing Set	267,885

**Table 6:** Contrast detection result.

Type		LSTM + Attention(LSAT)				CNN + LSTM(CNLS)				CNAT			
		Pre	Rec	MTDC	Response Time (min)	Pre	Rec	MTDC	Response Time (min)	Pre	Rec	MTDC	Response Time (min)
Botnet	Ares	0.99	0.99	81	141	0.99	0.99	69	166	0.96	0.99	97	20
	BYOB	0.91	0.68			0.85	0.7			0.81	0.97		
	Zeus	0.99	0.58			0.44	0.09			0.91	0.93		
	Mirai	0.99	0.99			0.99	0.99			0.91	0.99		
Net- work	SYN	0.99	0.99	99	140	0.99	0.99	99	156	0.99	0.99	99	22
	ACK	0.99	0.99			0.99	0.99			0.99	0.99		
	UDP	0.99	0.99			0.99	0.99			0.99	0.99		
LD- DoS	Slow Body	0.99	0.98	75	135	0.99	0.93	75	142	0.95	0.91	71	21
	Slow Headers	0.76	0.6			0.77	0.65			0.8	0.43		
	Slow Read	0.57	0.78			0.58	0.78			0.52	0.86		
	Shrew	0.99	0.66			0.99	0.67			0.99	0.66		
Appli- cation	CC	0.56	0.49	58	151	0.68	0.46	64	161	0.62	0.04	46	23
	HTTP Flood	0.8	0.97			0.81	0.97			0.92	0.99		
	HTTP Get	0.79	0.31			0.75	0.46			0.43	0.48		
	HTTP Post	0.83	0.57			0.85	0.67			0.67	0.35		
DR- DoS	NTP	0.96	0.99	99	113	0.94	0.99	98	171	0.99	0.99	97	18
	SNMP	0.95	0.99			0.98	0.98			0.93	0.99		
	SSDP	0.97	0.99			0.95	0.99			0.96	0.96		
	Chargen	0.89	0.99			0.91	0.99			0.88	0.99		
	Memcached	0.99	0.99			0.99	0.99			0.93	0.99		
	TFTP	0.97	0.99			0.95	0.99			0.95	0.94		

## Data Availability

The dataset created in this article can be found on github: https://github.com/liliMpro/source_dataset (accessed on 22 February 2022).
